# MDM2-Mediated Ubiquitination of RXRβ Contributes to Mitochondrial Damage and Related Inflammation in Atherosclerosis

**DOI:** 10.3390/ijms23105766

**Published:** 2022-05-21

**Authors:** Yi Zeng, Ji Cao, Chun-Xia Li, Chun-Yan Wang, Ruo-Man Wu, Xiao-Le Xu

**Affiliations:** Department of Pharmacology, Pharmacy College, Nantong University, Nantong 226001, China; zengyi1980@ntu.edu.cn (Y.Z.); cj2020ntu@163.com (J.C.); lcx258124186@163.com (C.-X.L.); wangcyxb@163.com (C.-Y.W.); wuruomanntu@163.com (R.-M.W.)

**Keywords:** murine double minute-2, retinoid X receptor beta, mitochondrial dysfunction, inflammation, atherosclerosis

## Abstract

A novel function of retinoid X receptor beta (RXRβ) in endothelial cells has been reported by us during the formation of atherosclerosis. Here, we extended the study to explore the cellular mechanisms of RXRβ protein stability regulation. In this study, we discovered that murine double minute-2 (MDM2) acts as an E3 ubiquitin ligase to target RXRβ for degradation. The result showed that MDM2 directly interacted with and regulated RXRβ protein stability. MDM2 promoted RXRβ poly-ubiquitination and degradation by proteasomes. Moreover, mutated MDM2 RING domain (C464A) or treatment with an MDM2 inhibitor targeting the RING domain of MDM2 lost the ability of MDM2 to regulate RXRβ protein expression and ubiquitination. Furthermore, treatment with MDM2 inhibitor alleviated oxidized low-density lipoprotein-induced mitochondrial damage, activation of TLR9/NF-κB and NLRP3/caspase-1 pathway and production of pro-inflammatory cytokines in endothelial cells. However, all these beneficial effects were reduced by the transfection of RXRβ siRNA. Moreover, pharmacological inhibition of MDM2 attenuated the development of atherosclerosis and reversed mitochondrial damage and related inflammation in the atherosclerotic process in LDLr^-/-^ mice, along with the increased RXRβ protein expression in the aorta. Therefore, our study uncovers a previously unknown ubiquitination pathway and suggests MDM2-mediated RXRβ ubiquitination as a new therapeutic target in atherosclerosis.

## 1. Introduction

Atherosclerosis is continuous crosstalk between the lipid metabolism and immune-inflammatory pathways. Intracellular lipid accumulation is a fundamental event in the pathogenesis of atherosclerosis at the cellular level. Reactive oxygen species (ROS) and reactive nitrogen species cause lipid peroxidation and low-density lipoprotein (LDL) oxi-dation [1]. Oxidized LDL (ox-LDL) then remains in the vascular intima. As a simple mon-olayer, vascular endothelium can respond to physicochemical stimuli. In addition to foam cell formation, ox-LDL contributes to atherogenesis through different mechanisms, in-cluding endothelial cell dysfunction which leads to increased expression or secretion of pro-inflammatory cytokines [2].

Retinoid X receptors (RXRs) belong to a nuclear receptor superfamily of ligand-dependent transcription factors whose effects on a diversity of cellular processes, including cell differentiation and proliferation, immune response, and lipid and glucose metabolism [3]. There are three RXR isotypes, which are classified as RXRα, RXRβ and RXRγ with distinct chromosomal locations. Recent advances reveal that RXRs may exert direct beneficial effects in cardiovascular diseases via homodimerization with itself or heterodimerization with other nuclear receptors [4,5]. Studies have shown that novel functions of RXRs in macrophage lipid homeostasis, inflammation activation, vascular protection and platelets contribute to the protective effect of RXRs in atherosclerosis [6,7,8,9,10,11,12,13]. However, most studies focused on RXRα. Recently, our group has firstly reported that RXRβ exerted protective effects against the ox-LDL-induced inflammation in human aortic endothelial cells (HAECs) through regulating peroxisome proliferator-activated receptor γ coactivator-1α (PGC1α)-dependent mitochondrial homeostasis [14]. Our further study found that protein expression of RXRβ was downregulated in the aorta of high-fat diet-fed low-density lipoprotein receptor-deficient (LDLr^-/-^) mice and ox-LDL-treated HAECs, but the mRNA level of RXRβ was not altered. Thus, these observations led us to study the mechanism of the degradation of RXRβ in endothelial cells.

Functioning as an E3 ubiquitin ligase, murine double minute 2 (MDM2) targets various substrates for mono- and/ or poly-ubiquitination, thereby regulating their activities, for instance, by controlling their stability, conformation, and/or localization by proteasome-dependent degradation [15]. Dysregulated MDM2 is implicated in cardiovascular impairments [16]. For example, MDM2 is overexpressed in human atherosclerotic lesions [17]. MDM2-mediated ubiquitination of histone deacetylase 1 enhances vascular calcification [18]. MDM2-induced ubiquitination of angiotensin-converting enzyme 2 contributes to the pathogenesis of pulmonary arterial hypertension [19]. Treatment with the small molecule MDM2 antagonist nutlin-3 reduced the intima area and the intimal/media area ratio after vascular injury by suppressing the proliferation of vascular smooth muscle cells and endothelial cells and lowering the level of inflammation [20]. Our previous study also showed that MDM2 contributed to ox-LDL-induced inflammation through modulation of mitochondrial damage in endothelial cells [21]. Therefore, based on our previous studies [14,21], we were encouraged to investigate whether MDM2 acts as an E3 ligase to regulate the stabilization of RXRβ and further illustrate the function of MDM2 in atherosclerosis.

Here, we report that MDM2 interacts with and modulates the stability of the RXRβ protein through the ubiquitin-proteasome system, which contributes to ox-LDL-induced mitochondrial damage and mitochondrial-related inflammation. Additionally, our results identify MDM2 as a potent target for atherosclerosis therapy.

## 2. Results

### 2.1. RXRβ Is Ubiquitinated in ox-LDL-Induced HAECs

**Figure 1:**
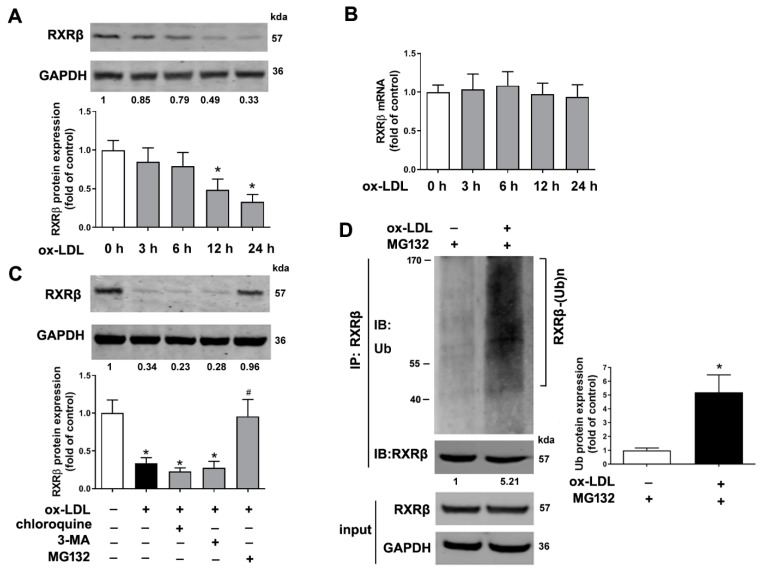
RXRβ is ubiquitinated in ox-LDL-induced HAECs. (**A**,**B**) protein and mRNA expression of RXRβ in HAECs which were incubated with 100 μg/mL ox-LDL at various times. (**C**) HAECs pretreated with 3-methyladenine (3-MA, 5 mM), chloroquine (25 μM), or MG132 (20 μM) for 1 h were incubated in the presence and absence of ox-LDL (100 μg/mL) for 24 h. Then, protein expression of RXRβ was determined. (**D**) The immunoprecipitation results showed the ubiquitination of RXRβ in HAECs under ox-LDL (100 μg/mL) for 24 h in the presence of MG132 (20 μM). Results are presented as the mean ± SD. *n* = 6. * *p* < 0.05 vs. control group, # *p* < 0.05 vs. ox-LDL alone group.

The result of Figure 1A showed that the protein expression of RXRβ reached the lowest level at 24 h after 100 μg/mL ox-LDL treatment. However, the present study showed that the mRNA level of RXRβ was not altered compared with the control group (Figure 1B). We next studied the mechanism of the degradation of RXRβ in ox-LDL-treated HAECs. As shown in Figure 1C, ox-LDL-induced reduction of RXRβ was significantly attenuated by treatment with MG132, a proteasome inhibitor, but not by treatment with the inhibitor of autophagy (3-methyladenine, 3-MA) or lysosome (chloroquine). Moreover, by using an immunoprecipitation-based ubiquitination assay, our data demonstrated that in the presence of MG132, which was added to inhibit the degradation of RXRβ, RXRβ was heavily ubiquitinated in HAECs exposed to ox-LDL (Figure 1D).

### 2.2. MDM2 Can Affect the Stability of RXRβ

**Figure 2:**
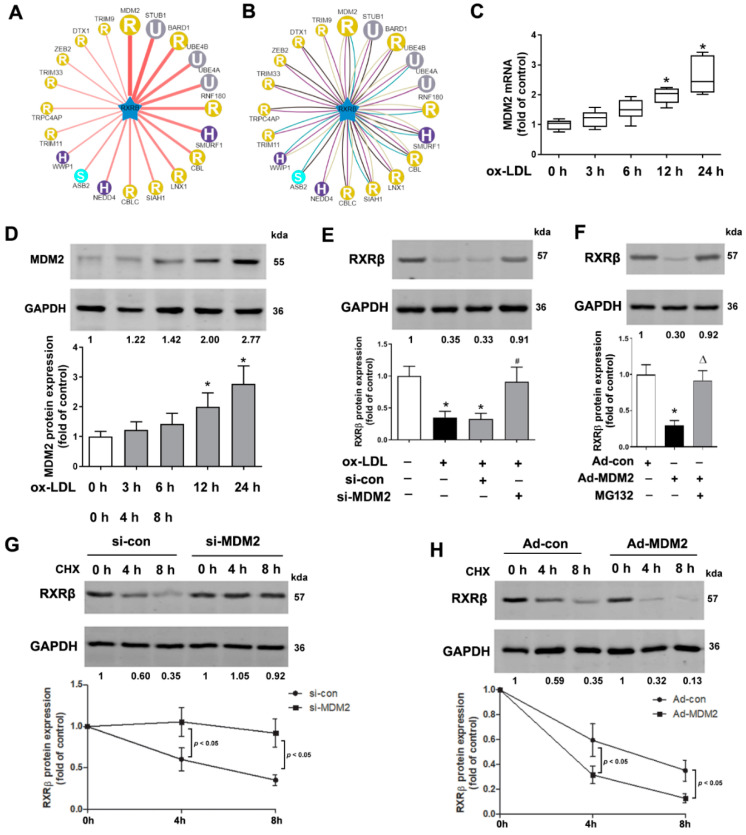
MDM2 can affect the stability of RXRβ. (**A**,**B**) MDM2 is predicted as the specific E3 ligase of RXRβ by the UbiBrowser database. In the confidence mode (**A**) and evidence mode (**B**), a node is positioned in the center of the canvas showing the putative substrates, surrounded by nodes revealing predicted E3 ligases. The node colors and characters denote the E3 ligase type with the edge width and node size representing the confidence score. (**C**,**D**) mRNA and protein expression of MDM2 in HAECs which were incubated with 100 μg/mL ox-LDL at various times. (**E**) HAECs were incubated with control siRNA (si-con, 100 nM) or MDM2 siRNA (si-MDM2, 100 nM) for 24 h and then with 100 μg/mL ox-LDL for additional 24 h. Then, the protein expression of RXRβ in HAECs was determined. (**F**) HAECs were incubated with a non-targeting control vector (Ad-con) or MDM2-adenoviral vector (Ad-MDM2) in the presence and absence of MG132 (20 μM) for 24 h. Then, the protein expression of RXRβ in HAECs was determined. (**G**,**H**) Western blotting in lysates from HAECs transfected with si-MDM2, si-con, Ad-MDM2 or Ad-con in the presence of cycloheximide (100 μg/mL) for up to 8 h. The results of (**C**) were represented as median, 10th percentile, 90th percentile, and minimum and maximum values. The results of (**D**–**H**) are presented as the mean ± SD. *n* = 6. * *p* < 0.05 vs. control group, # *p* < 0.05 vs. ox-LDL alone group, Δ *p* < 0.05 vs. Ad-MDM2 group.

According to the UbiBrowser database (http://ubibrowser.ncpsb.org/, accessed on 11 April 2022), which is a resource of known and predicted human ubiquitin ligase (E3)-substrate interaction network, MDM2 is predicted with the highest confidence as a primary E3 ligase for RXRβ (Figure 2A,B). Consistently, the protein and mRNA levels of MDM2 were both increased in ox-LDL-treated HAECs time-dependently accompanied by downregulation of the RXRβ protein expression (Figure 2C,D). We next explored whether MDM2 regulates RXRβ stability. The efficiency of MDM2 knockdown or overexpression in HAECs was confirmed by our previous study [21]. As shown in Figure 2E, MDM2 knockdown by siRNA increased RXRβ protein expression in ox-LDL-treated HAECs. By contrast, MDM2 overexpression in HAECs decreased the RXRβ protein level, which was abolished by the proteasome inhibitor MG132 (Figure 2F). These results strongly suggested RXRβ as a new substrate of MDM2, indicating that MDM2 may degrade RXRβ in some way and this degradation likely occurs via the proteasome pathway. We then tested MDM2 for its effect on RXRβ protein stability following treatment with cycloheximide, an inhibitor of protein synthesis. Our data showed that MDM2 knockdown prolonged, but MDM2 overexpression shorted the half-life of endogenous RXRβ in HAECs (Figure 2G,H).

### 2.3. MDM2 Mediates the Poly-Ubiquitination of RXRβ

**Figure 3:**
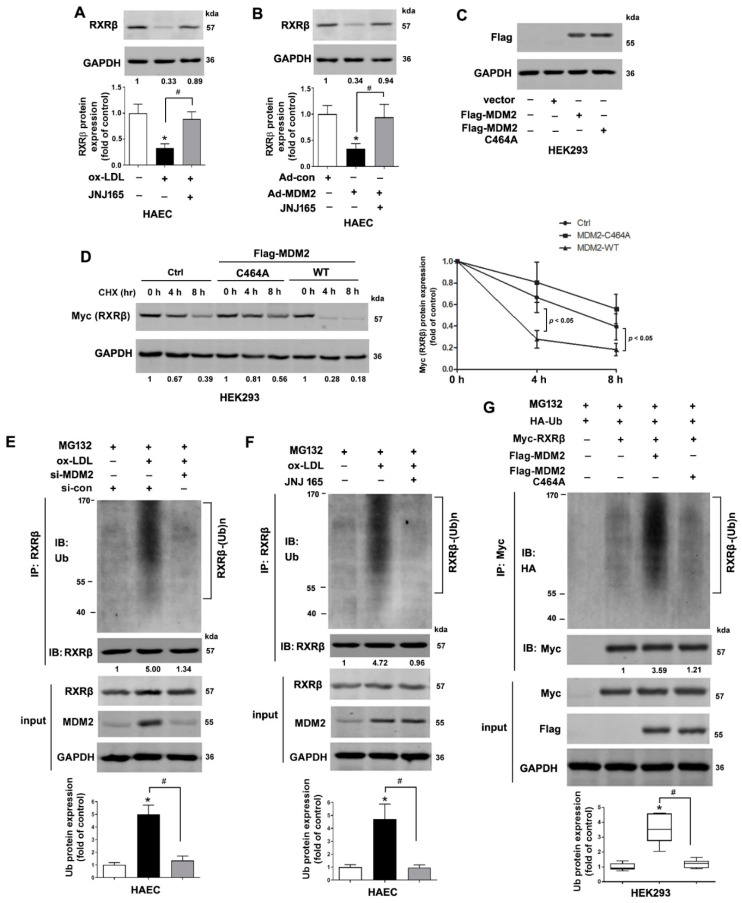
MDM2 mediates the poly-ubiquitination of RXRβ. (**A**) HAECs pretreated with JNJ-165 (10 μM) or vehicle for 1 h were incubated in the presence and absence of ox-LDL (100 μg/mL) for 24 h. Then, protein expression of RXRβ was determined. (**B**) HAECs were incubated with a non-targeting control vector (Ad-con) or MDM2-adenoviral vector (Ad-MDM2) in the presence and absence of JNJ-165 (10 μM) for 24 h. Then, the protein expression of RXRβ in HAECs was determined. (**C**) HEK293 cells were transfected with Flag-tagged wild-type (WT) MDM2 or Flag-tagged MDM2 C464A mutant plasmids for 48 h. The efficiency was confirmed by Western blot analysis. (**D**) Western blot analysis of Myc (RXRβ) in HEK293 cells transfected with Flag-tagged WT MDM2 or Flag-tagged MDM2 C464A mutant plasmids together with Myc-tagged-RXRβ plasmids, then treated with cycloheximide (100 μg/mL) for up to 8 h. (**E**) The immunoprecipitation results showed the ubiquitination of RXRβ in MDM2 siRNA transfected HAECs under ox-LDL (100 μg/mL) for 24 h in the presence of MG132 (20 μM). (**F**) The immunoprecipitation results showed the ubiquitination of RXRβ in JNJ-165 (10 μM) pretreated HAECs under ox-LDL (100 μg/mL) for 24 h in the presence of MG132 (20 μM). (**G**) Myc-RXRβ and HA-Ub with either Flag-MDM2 or Flag-MDM2 C464A mutant plasmids were transfected and maintained for 48 h in HEK293 cells. Cells were treated with MG132 for 4 h before collecting. The cell lysates were immunoprecipitated with Myc and immunoblotted with HA. The results of (**A**–**F**) are presented as the mean ± SD. The results of (**G**) were represented as median, 10th percentile, 90th percentile, and minimum and maximum values. *n* = 6. * *p* < 0.05 vs. control group. # *p* < 0.05.

As a RING-type E3 ubiquitin ligase, the ubiquitinase catalytic domain, RING domain, of MDM2 is indispensable for its ubiquitination activity. Deletion of the RING domain of MDM2 results in the loss of E3 ligase activity [22,23]. As shown in Figure 3A,B, MDM2 inhibition by JNJ-165, an agent targeting the RING domain of MDM2 [19], increased RXRβ protein expression in ox-LDL-treated or Ad-MDM2-transfected HAECs. Consistently, overexpression of the loss-of-function mutant MDM2-C464A that carries a RING finger mutant with cysteine changed to alanine did not affect the protein half-life of RXRβ (Figure 3C,D) in HEK293 cells. Next, we investigated whether MDM2 ubiquitinates RXRβ and therefore affects RXRβ stability. Treatment with JNJ-165 or transfected with MDM2 siRNA significantly attenuated the poly-ubiquitination of RXRβ in ox-LDL-treated HAECs (Figure 3E,F). Consistently, transfection of wild-type MDM2 to HEK293 cells enhanced the ubiquitination of RXRβ. However, transfection of mutant MDM2 (C464A) that lacked the RING domain for E3 ligase activity failed to do so (Figure 3G).

### 2.4. MDM2 Interacts with RXRβ

**Figure 4:**
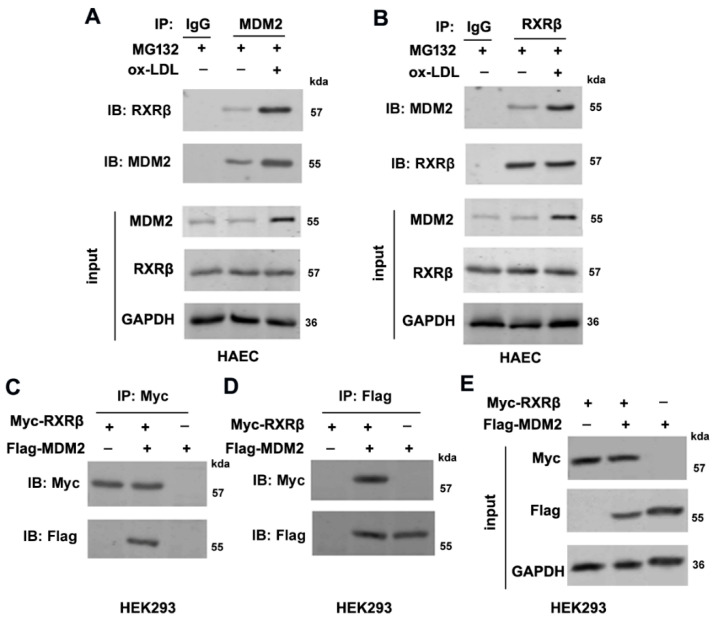
MDM2 interacts with RXRβ. (**A**,**B**) HAECs were incubated with 100 μg/mL ox-LDL in the presence of MG132 (20 μM) for 24 h, and cell lysates were then immunoprecipitated with anti-MDM2 or anti-RXRβ or negative control IgG antibodies followed by immunoblotting with an anti-MDM2 or anti-RXRβ antibody to verify endogenous interaction between MDM2 and RXRβ. The input protein was immunoblotted with antibodies against RXRβ, MDM2, or GAPDH. (**C**–**E**) Transfection of HEK293 cells with Myc-RXRβ together with Flag-MDM2 for 48 h, and cell lysates were then immunoprecipitated with anti-Myc or anti-Flag antibody followed by immunoblotting with an anti-Myc or anti-Flag antibody to verify the exogenous interaction between MDM2 and RXRβ. The input protein was immunoblotted with antibodies against Myc, Flag, or GAPDH.

Next, we examined whether RXRβ and MDM2 could physically interact. Endogenously expressed MDM2 and RXRβ in HAECs were reciprocally immuno-precipitated both with or without ox-LDL (Figure 4A,B). Next, we overexpressed Flag-MDM2 and Myc-RXRβ in HEK293 cells, and this exogenous co-immunoprecipitation verified the exogenous interaction between Flag-MDM2 and Myc-RXRβ (Figure 4C–E).

### 2.5. MDM2 Inhibitor JNJ-165 Provides Protection against ox-LDL-Induced Mitochondrial Damage via RXRβ

**Figure 5:**
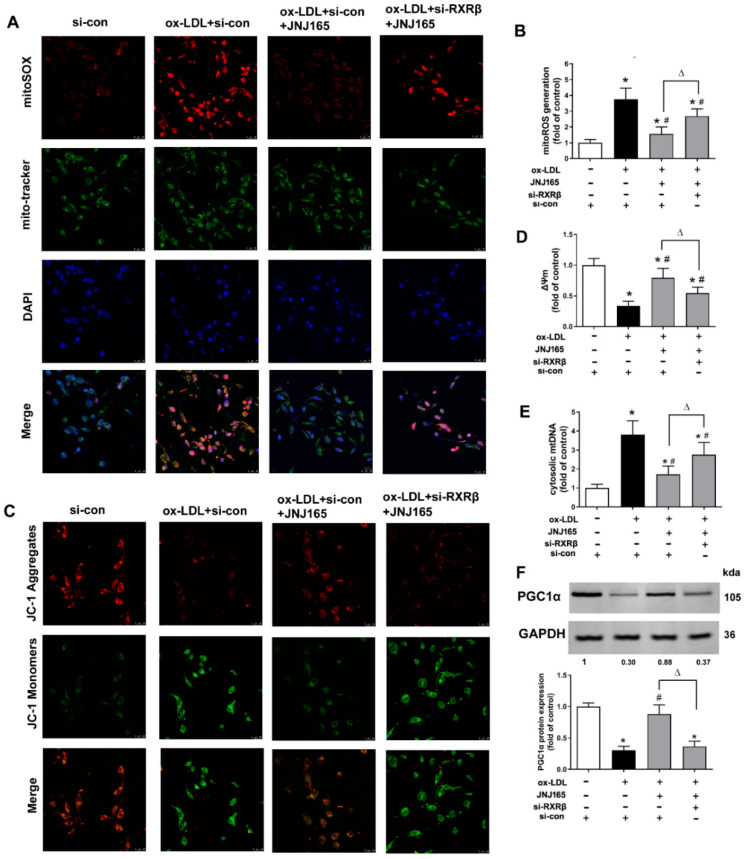
MDM2 inhibitor JNJ-165 provides protection against ox-LDL-induced mitochondrial damage via RXRβ. HAECs were transfected with RXRβ siRNA (si-RXRβ) or si-con for 24 h and then treated with or without JNJ-165 (10 μM). After 1 h, the cells were stimulated with 100 μg/mL ox-LDL for an additional 24 h. The mitoROS levels were determined based on the mitoSOX fluorescence by fluorescence microscope (**A**) and fluorescence microplate reader (**B**). The ΔΨm levels were determined based on the fluoroprobe JC-1 by fluorescence microscope (**C**) and fluorescence microplate reader (**D**). (**E**) The content of cytosolic mtDNA. (**F**) The protein expression of PGC1α. Results are presented as the mean ± SD. *n* = 6. * *p* < 0.05 vs. si-con with no treatment, # *p* < 0.05 vs. ox-LDL + si-con group, Δ *p* < 0.05.

As shown in Figure 5A–E, ox-LDL caused significant mitochondria damage indicated as the increased mtROS production, decreased membrane potential (ΔΨm) and enhanced mtDNA release in HAECs. MDM2 inhibitor JNJ-165 alleviated such mitochondrial damage. However, transfection of RXRβ siRNA abrogated the alleviation of mitochondrial damage treated with JNJ-165. PGC1α is a well-known master regulator of mitochondrial biogenesis. Furthermore, our previous study showed that PGC1α is one target gene of RXRβ in HAECs [14]. As shown in Figure 5F, JNJ-165 increased the protein expression of PGC1α in ox-LDL treated endothelial cells, which was also abrogated by the transfection of RXRβ siRNA (Figure 5F).

### 2.6. MDM2 Inhibitor JNJ-165 Provides Protection against ox-LDL-Induced Mitochondrial Related Inflammation via RXRβ

**Figure 6:**
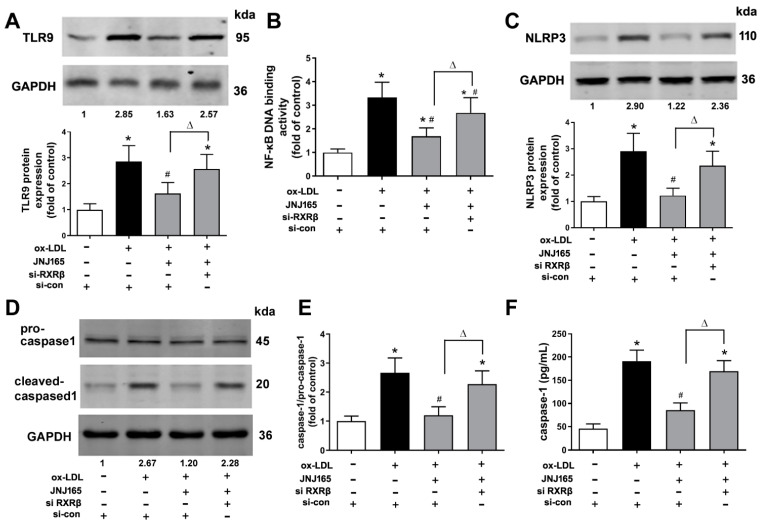

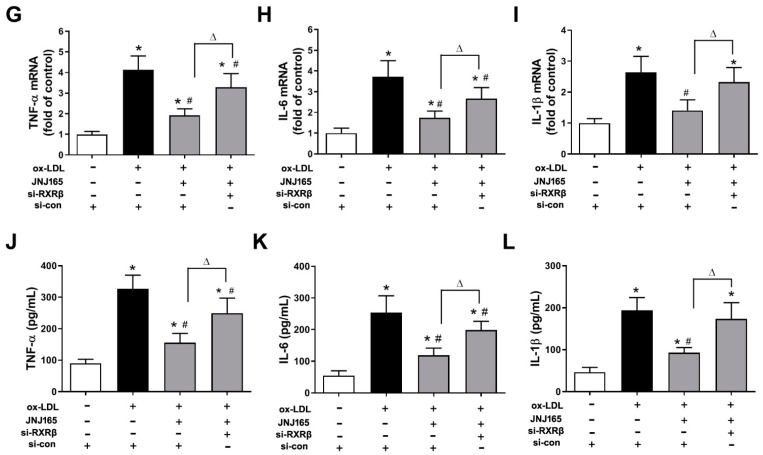
MDM2 inhibitor JNJ-165 provides protection against ox-LDL-induced mitochondrial-related inflammation via RXRβ. HAECs were transfected with RXRβ siRNA (si-RXRβ) or si-con for 24 h and then treated with or without JNJ-165 (10 μM). After 1 h, the cells were stimulated with 100 μg/mL ox-LDL for an additional 24 h. (**A**) The protein expression of TLR9. (**B**) NF-κB p65 DNA binding activity. (**C**) The protein expression of NLRP3. (**D**,**E**) The protein expression of caspase-1. (**F**) The level of caspase-1 p20 in culture supernatants. (**G**–**I**) The mRNA expression of TNF-α (**G**), IL-6 (**H**) and IL-1β (**I**). (**J**–**L**) The secretion of TNF-α (**J**), IL-6 (**K**) and IL-1β (**L**). Results are presented as the mean ± SD. *n* = 6. * *p* < 0.05 vs. si-con with no treatment, # *p* < 0.05 vs. ox-LDL + si-con group, Δ *p* < 0.05.

Mitochondrial homeostasis is closely associated with the activation of Toll-like receptor 9 (TLR9)/NF-κB and NLRP3/caspase-1 inflammasome pathway [24]. Our previous study has demonstrated that upregulation of MDM2 protein expression was involved in ox-LDL-induced mitochondrial damage, which subsequently mediated the activations of TLR9/NF-κB and NLRP3/caspase-1 inflammasome to further produce pro-inflammatory cytokines [21]. The present results further showed that MDM2 inhibitor JNJ-165 significantly decreased the protein expression of TLR9 and NF-κB p65 DNA binding activity. In addition, JNJ-165 significantly reduced the protein expression of NLRP3, the ratio of caspase-1 p20 to pro-caspase-1 in cell lysate and the secretion of bioactive caspase-1 p20 in culture supernatants in ox-LDL treated HAECs. However, all these decreases were abrogated by the transfection of RXRβ siRNA (Figure 6A–F). Furthermore, JNJ-165 significantly decreased the levels of mRNA expression and secretion of TNF-α, IL-6 and IL-1β in ox-LDL treated HAECs, which were all significantly reversed by the transfection of RXRβ siRNA (Figure 6G–L).

### 2.7. MDM2 Inhibitor JNJ-165 Provides Protection against ox-LDL-Induced Mitochondrial Damage and Related Inflammation Independent of p53

**Figure 7:**
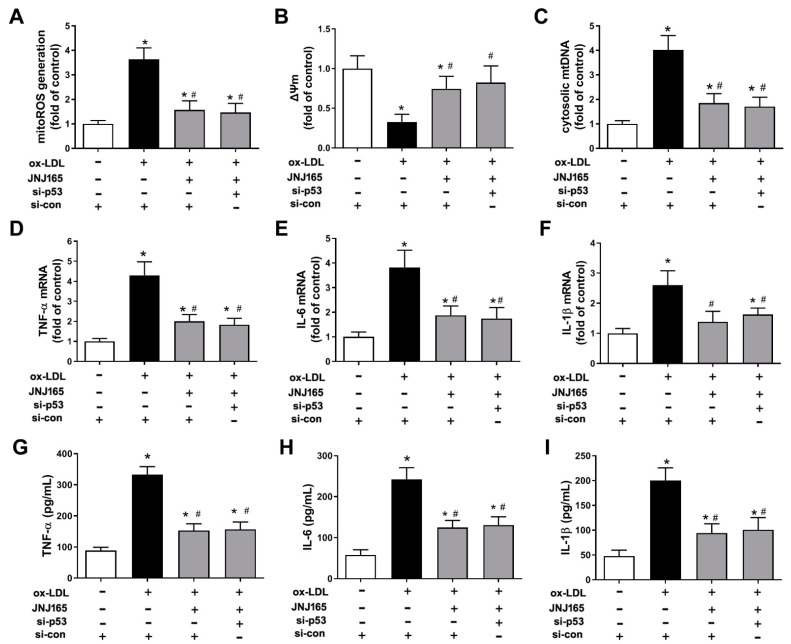
MDM2 inhibitor JNJ-165 provides protection against ox-LDL-induced mitochondrial damage and related inflammation independent of p53. For (**A**–**I**), HAECs were transfected with p53 siRNA (si-p53) or si-con for 24 h and then treated with or without JNJ-165 (10 μM). After 1 h, the cells were stimulated with 100 μg/mL ox-LDL for an additional 24 h. (**A**) mitoROS. (**B**) ΔΨm. (**C**) mtDNA. (**D**–**F**) The mRNA expression of TNF-α (**D**), IL-6 (**E**) and IL-1β (**F**). (**G**–**I**) The secretion of TNF-α (**G**), IL-6 (**H**) and IL-1β (**I**). Results are presented as the mean ± SD. *n* = 6. * *p* < 0.05 vs. si-con with no treatment, # *p* < 0.05 vs. ox-LDL + si-con group.

Since MDM2 is well known as an E3 ubiquitin-protein ligase for tumor-suppressor p53, whether p53 was involved in the above protection of JNJ-165 was determined. As expected, JNJ-165 treatment increased the protein levels of p53, presumably by inhibiting MDM2-mediated p53 degradation (Supplemental Figure S1). However, the protective effects of JNJ-165 on ox-LDL-induced mitochondrial damage and mitochondrial-related inflammation remained unaffected by transfection of p53 siRNA (Figure 7), suggesting that JNJ-165 treatment suppressed ox-LDL-induced mitochondrial damage and mitochondrial related inflammation in HAECs most likely independent of p53. The above results suggest that MDM2 inhibitor JNJ-165 provides protection against ox-LDL-induced mitochondrial damage and related inflammation partially through the protein RXRβ.

### 2.8. MDM2 Inhibition Alleviates Atherosclerotic Lesion Formation and RXRβ Protein Expression in HFD-Fed LDLr^-/-^ Mice

**Figure 8:**
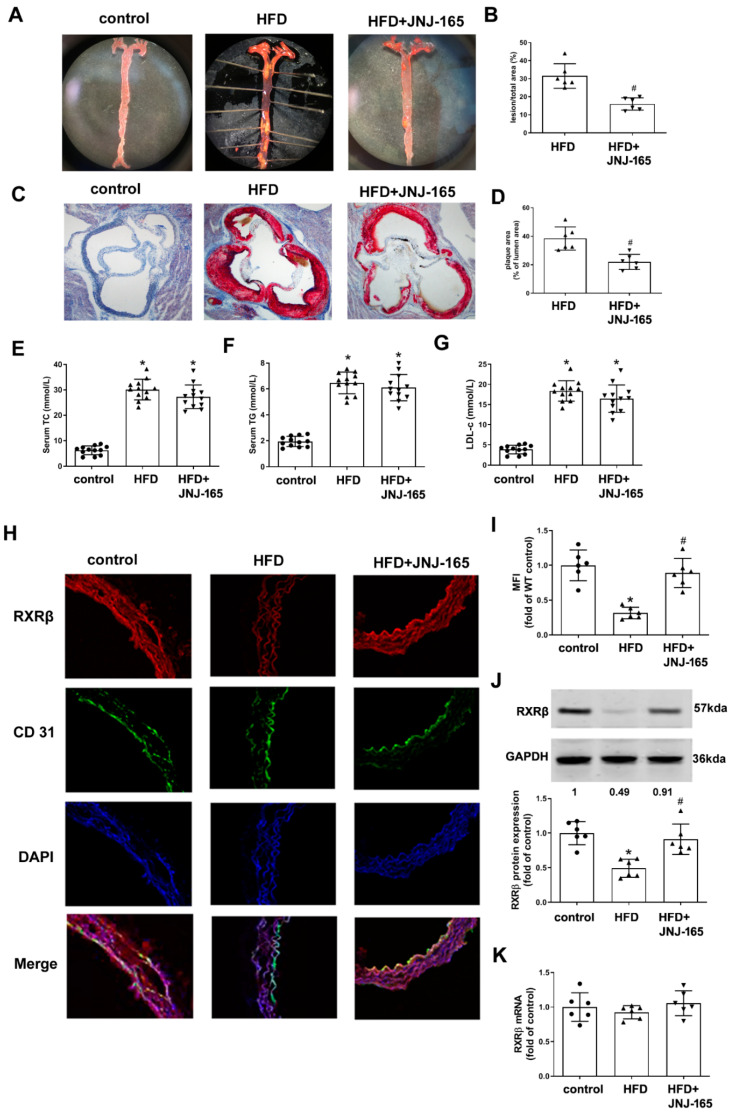
MDM2 inhibition alleviates atherosclerotic lesion formation and RXRβ protein expression in HFD-fed LDLr^-/-^ mice. (**A**,**B**) Representative images and quantification of Oil Red O staining of en face preparations of aortas. *n* = 6. (**C**,**D**) Representative images and quantification of aortic root sections stained with Oil Red O. *n* = 6. (**E**–**G**) Serum levels of TC, TG and LDL-c. *n* = 12. (**H**) Representative images of thoracic aorta cross-sections stained with anti-RXRβ antibody and CD31, respectively. (**I**) Quantification of fluorescence intensity of RXRβ staining. *n* = 6. (**J**) Protein expression of RXRβ in the aorta. n = 6. (**K**) mRNA expression of RXRβ in the aorta. *n* = 6. Results are presented as the mean ± SD. * *p* < 0.05 vs. control group. # *p* < 0.05 vs. HFD group. The individual data points are shown as the circles, the normal or upside down triangles on top of the corresponding bars.

Our previous studies have shown that MDM2 protein expression was upregulated, whereas RXRβ protein expression was downregulated in the aortas of LDLr^-/-^ mice fed a high-fat diet [14,21]. This time we examined whether blockade of MDM2 activity by JNJ-165 could result in the prevention of atherosclerosis in atherosclerotic mice. Our results showed that JNJ-165 administration significantly inhibited atherosclerotic lesion formation in the *en* face prepared aorta and aortic root compared to the model group (Figure 8A–D). However, JNJ-165 did not affect lipid profiles (Figure 8E–G), indicating that the reduction of lesions by JNJ-165 is unrelated to its effect on lipid homeostasis. Immunofluorescence analysis for RXRβ showed that the expression of RXRβ in CD31 co-stained cells (endothelial cells) was restored by administration of JNJ-165 (Figure 8H,I). The result of the Western blot also confirmed that the protein expression of RXRβ was significantly increased in the aortas of JNJ-165 treatment mice (Figure 8J). However, the mRNA level of RXRβ was not altered in all groups (Figure 8K).

### 2.9. MDM2 Inhibition Alleviates Mitochondrial Damage and Related Inflammation in HFD-Fed LDLr^-/-^ Mice

**Figure 9:**
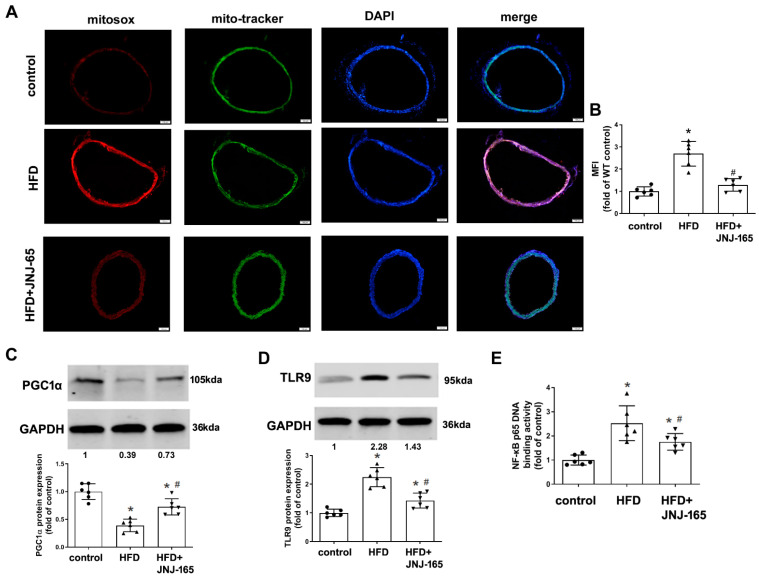

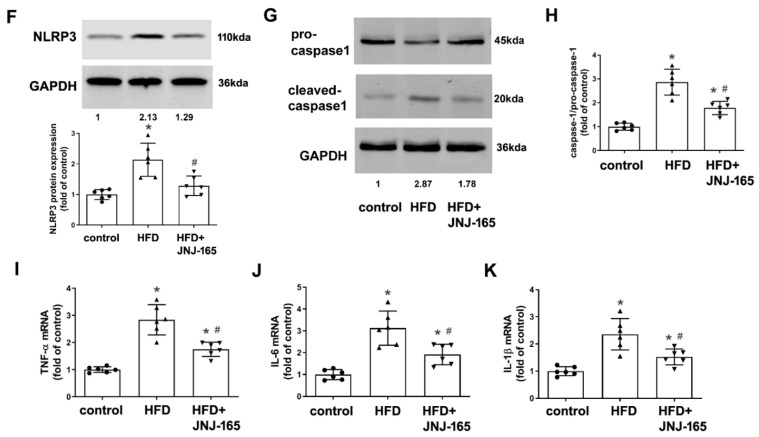
MDM2 inhibition alleviates mitochondrial damage and related inflammation in HFD-fed LDLr^-/-^ mice. (**A**,**B**) Representative images and quantification of thoracic aorta cross-sections stained with mitoSOX Red. (**C**) The protein expression of PGC1α. (**D**) The protein expression of TLR9. (**E**) NF-κB p65 DNA binding activity. (**F**) The protein expression of NLRP3 in the aorta. (**G**,**H**) The protein expression of caspase-1. (**I**–**K**) mRNA expression of TNF-α (**I**), IL-6 (**J**) and IL-1β (**K**) in the aorta. Results are presented as the mean ± SD. (*n* = 6). * *p* < 0.05 vs. control group. # *p* < 0.05 vs. HFD group. The individual data points are shown as the circles, the normal or upside down triangles on top of the corresponding bars.

Administration of JNJ-165 significantly reduced mtROS production, but increased protein expression of PGC1α in the aortas of HFD-fed LDLr^-/-^ mice (Figure 9A–C). In addition, our results showed that administration of JNJ-165 to HFD-fed LDLr^-/-^ mice significantly decreased TLR9 protein expression and NF-κB p65 DNA binding activity (Figure 9D,E). Administration of JNJ-165 also significantly decreased the protein expression of NLRP3 and the ratio of caspase-1 p20 to pro-caspase-1 in the aortas (Figure 9F–H). Moreover, JNJ-165 administration significantly decreased the levels of mRNA expression of TNF-α, IL-6 and IL-1β in the aortas of HFD-fed LDLr^-/-^ mice (Figure 9I–K). These results suggest that pharmacological inhibition of MDM2 may alleviate mitochondrial damage and related inflammation in the pathological progression of atherosclerosis.

## 3. Discussion

The ubiquitin-proteasome system has emerged as an important regulatory mechanism of nuclear receptor function [25]. Both nuclear receptors and their co-regulators are targeted to the ubiquitin-proteasome system for protein stability. We have previously shown that RXRβ exerts protective effects against the ox-LDL-induced inflammation in endothelial cells through regulating PGC1α-dependent mitochondrial homeostasis [14]. However, the molecule mechanism and regulation of RXRβ in endothelial cells are still unclear. Here, we extended the study to explore the cellular mechanisms of RXRβ protein stability regulation. The present study demonstrates for the first time that MDM2 can directly interact with and regulate RXRβ protein stability. MDM2 promotes RXRβ poly-ubiquitination and degradation by proteasomes. MDM2 appears to function as a ubiquitin E3 ligase in this process, since the MDM2 RING domain mutant or inhibition reduces the ubiquitination of RXRβ. Furthermore, the present study for the first time shows that pharmacological inhibition of MDM2 (which increased protein expression of RXRβ) attenuates the development of atherosclerosis and reverses mitochondrial damage and the related inflammation in this process.

In the current study, we found ubiquitination-proteasome-pathway mediated degradation of RXRβ. Consistent with our finding, ubiquitination has been reported as an important determinant of RXRα half-life [4]. As a posttranslational modification, ubiquitination alters the stability, conformation, or localization of the target protein. In this process, ubiquitin (Ub) is attached to one or more lysine residues of a substrate protein and forms mono- or poly-Ub chains. Ub-ligase (E3) determines substrate specificity in the ubiquitin proteasomal system [26]. According to the UbiBrowser database, MDM2 is predicted with the highest confidence as a primary E3 ligase for RXRβ. MDM2 is well known as an E3 ubiquitin-protein ligase for tumor-suppressor p53. It has also been found to mediate the ubiquitination of many other substrates such as Rb [27], ACE2 [19], HDAC1 [18], PKCβII [28], and PPARα [25], leading to their degradation by the proteasome. As expected, we find that RXRβ is a newly discovered substrate of MDM2. Our results showed that MDM2 negatively regulated the RXRβ protein expression and degraded RXRβ through the proteasome pathway. RXRβ was modified by MDM2-dependent poly-ubiquitination. Mutated MDM2 RING domain (C464A) or treatment with JNJ-165, an agent targeting the RING domain of MDM2, lost the ability of MDM2 to regulate RXRβ ubiquitination, suggesting that MDM2 promotes the poly-ubiquitination of RXRβ relying on the catalytic activity of its RING finger domain. Furthermore, by protein–protein interaction experiments, we found that MDM2 directly interacted with RXRβ. All these findings suggest that MDM2 may function as a ubiquitin E3 ligase for RXRβ.

MDM2 is involved in many important biological processes. In addition to its well-known tumorigenic characteristics by impacting p53, MDM2 is also reported to be involved in the pathophysiological events of the cardiovascular system [16,17,18,19,20]. Our previous study has indicated that MDM2 contributes to ox-LDL-induced activation of the TLR9/NF-κB pathway and NLRP3/caspase-1 inflammasome through modulation of mitochondrial damage in endothelial cells [21]. Therefore, we next wanted to know whether such adverse effect of MDM2 in endothelial cells is ascribed to RXRβ degradation. Consistent with our previous study using siRNA for MDM2 downregulation [21], the present study for the first time showed that MDM2 inhibitor JNJ-165 also alleviated ox-LDL-induced mitochondrial damage indicated as the decreased generation of mtROS, increased ΔΨm and decreased mtDNA release. Moreover, JNJ-165 inhibited ox-LDL-induced activation of TLR9/NF-κB and NLRP3/caspase-1 pathway and production of pro-inflammatory cytokines. All these beneficial effects of JNJ-165 were reduced by the transfection of RXRβ siRNA, but not p53 siRNA. In line with our results, there is a notion that beyond the primary function of MDM2 to keep p53 under control, MDM2 has functions that are independent of p53 in vascular cells such as endothelial cells, vascular smooth cells and cardiomyocytes [16]. Moreover, the in vivo experiment in the present study for the first time showed that MDM2 inhibitor JNJ-165 alleviated the development of atherosclerosis and reversed mitochondrial damage and related inflammation in this process, along with the increased RXRβ protein expression in the aorta of mice. These findings suggest the therapeutic effect of MDM2 inhibitors on atherosclerosis, if any, would be attributable, at least in part, to their inhibition of the MDM2-RXRβ axis.

In the last few years, there has been growing interest in the relationship between cardiovascular disease and cancer [29]. Additionally, some anticancer drugs have been used in clinical trials of cardiovascular disease [19,30]. Since MDM2 inhibitors are now being under extensive investigation for the treatment of cancer by recent research works, application of those drugs for the treatment of cardiovascular disease may be anticipated [31]. Indeed, JNJ-165, used in the present study, has been suggested to exert a protective effect on the development of pulmonary arterial hypertension [19]. Nutlin-3, the small molecule MDM2 antagonist, can reduce the intima area and the intimal/media area ratio after vascular injury [20]. Treatment with MDM2 inhibitor RG 7112 prevents vascular calcification [18]. Contrary to the vascular protective effect of MDM2 inhibitors, it is reported that cardiac-specific ablation of MDM2 leads to broad mitochondrial deficiency and reduced expression of MDM2 in the cardiomyocytes may facilitate cardiomyocyte hypertrophy and cardiac hypertrophy. These seemingly contradictory findings have been explained by the plasticity of MDM2 including the tissue and cell type-specific differences in MDM2 function [31]. MDM2 is considered a hub protein due to its capacity to interact with more than 100 different partners and the list is continuously increasing [15]. Under different conditions, MDM2 interacts with different substrates to provide a key intermediary role in different signaling pathways [15,16]. For example, several studies uncovered the role of MDM2 in mitochondrial bioenergetics independently of p53 leading to decreased mitochondrial respiration, marked oxidative stress and DNA damage [32,33,34]. Thus, the present study may serve as a new example to highlight the diverse role of MDM2 as a central hub in the cardiovascular system.

Despite the new findings reported here, the potential limitations of this study should be noted. First, although JNJ-165 treatment suppressed ox-LDL-induced mitochondrial damage and inflammation in HAECs independent of p53, we cannot exclude the possible role of p53 in the protective effect of JNJ-165 against atherosclerosis by other mechanisms in different aortic cells, since this gene is involved in many signaling pathways that cause cell senescence, apoptosis and cell cycle arrest in the pathogenesis of atherosclerosis [35]. Thus, further investigations are needed. Second, a study to evaluate the safety and tolerability of JNJ-165 is needed since there could be potential deleterious/toxic effects occurring from systemic MDM2 inhibition via JNJ-165, especially on the cardiomyocytes.

## 4. Materials and Methods

An expanded Materials and Methods section is available in the Supplemental Materials and Methods. In brief, the 8-week-old male LDLr^-/-^ mice were randomly separated into control, high-fat diet (HFD) and JNJ-26854165 (JNJ-165) -treatment groups. The control group was fed a standard chow diet for 12 weeks. In the HFD group, the mice were fed a high-fat diet containing 0.21% cholesterol and 21% fat for 12 weeks continuously. In the JNJ-165-treatment group, mice were administered the same HFD for 12 weeks and dosed daily via oral gavage with 20 mg/kg/day JNJ-165 by weight for the last 4 weeks. JNJ-165 was suspended in 0.5% carboxymethyl cellulose. Mice in the control and HFD-only group received the same volume of 0.5% carboxymethyl cellulose vehicle gastrically. At the end of the 12 weeks, all mice were sacrificed under pentobarbital sodium anesthesia (50 mg/kg, *i.p.*). Aorta tissues were collected for further analysis.

Human aortic endothelial cells (HAECs) were grown in the medium supplemented with endothelial cell growth supplement and 10% fetal bovine serum (FBS). HAECs at 3 to 8 passages were used in the study. HEK293 cells were grown in DMEM supplemented with 10% FBS. When transfected with two or more plasmids, HAECs have relatively low transfection rates. In these experiments, HEK293 cells were used for co-transfection. HEK293 cells were also used to detect the exogenous interaction between MDM2 and RXRβ.

For transfection, MDM2 siRNA and p53 siRNA were purchased from GenePharma RNAi company (Shanghai, China). RXRβ siRNA and MDM2 adenovirus vector (Ad-MDM2) were designed and synthesized by Hanbio Biotechnology Co., Ltd. (Shanghai, China). HA-tagged ubiquitin, Myc-tagged-RXRβ, Flag-tagged MDM2 and Flag-tagged MDM2 C464A mutant plasmids were constructed by Hanbio Biotechnology Co., Ltd. (Shanghai, China). For siRNA transfection, cells were transfected with MDM2 siRNA, RXRβ siRNA or p53 siRNA for 24 h at a final concentration of 100 nM as we described previously [14,21]. To overexpression MDM2, cells were transfected with MDM2 adenovirus vector. MDM2 adenovirus vector diluted to MOI value of 100 was added to the culture medium for 24 h as we described previously [21]. For plasmids transfection, all plasmids were transfected with Lipofectamine 3000 for 48 h.

The level of inflammatory cytokines tumor necrosis factor-α (TNF-α), interleukin 6 (IL-6) and interleukin-1β (IL-1β) in cell culture supernatants were detected by ELISA kits. NF-κB (p65) transcription factor DNA-binding was detected using a transcription factor assay kit that we have described previously [14,21].

Mitochondria-derived reactive oxygen species (mtROS), mitochondrial membrane potential (ΔΨm) and cytosolic mitochondrial DNA (mtDNA) were detected in HAECs in line with our previous reports [14,21]. The representative images of mtROS and ΔΨm were visualized with a fluorescence microscope (Olympus, Tokyo, Japan).

Quantitative real-time PCR, Western blot and immunoprecipitation analysis were determined as we described previously [14,21,36,37,38].

### Statistical Analysis

Data analyses were performed using IBM SPSS Statistics 26 (Chicago, IL, USA) or Graphpad 8.0 (GraphPad Software, San Diego, CA, USA). Before statistical testing, data were explored to assess their pattern of distribution. For data with normal distribution and equal variances, an unpaired Student’s *t*-test was used to compare two groups and one-way ANOVA with the Newman–Keuls test for multiple groups. The results were represented as mean ± S.D. For data with unequal variances, nonparametric Kruskal–Wallis test with Dunn’s multiple comparison post-test for multiple groups. The results were represented as median, 10th percentile, 90th percentile, and minimum and maximum values. *p* < 0.05 was considered statistically significant.

## 5. Conclusions

**Figure 10:**
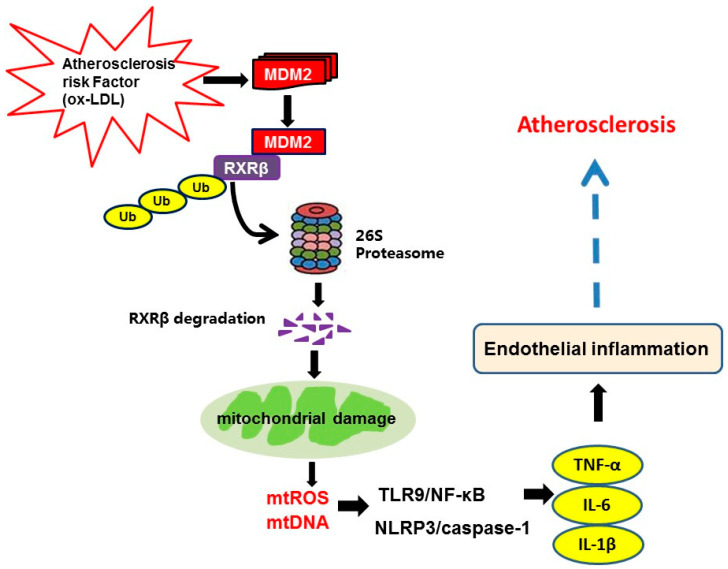
Illustration of the mechanism by which MDM2-mediated ubiquitination of RXRβ contributes to atherosclerosis. Under conditions that lead to atherosclerosis, such as upon prolonged exposure to ox-LDL in endothelial cells, the expression of MDM2 is induced, and MDM2 functions as a ubiquitin E3 ligase for RXRβ. MDM2 promotes RXRβ poly-ubiquitination and degradation by proteasomes, which contributes to mitochondrial damage. Subsequently, the elevated amounts of mtROS and release of mtDNA active TLR9/NF-κB pathway and NLRP3/caspase-1 inflammasome, which further produces pro-inflammatory cytokines TNF-α, IL-6 and IL-1β in endothelial cells and finally promotes the progression of atherosclerosis.

In summary, the present study elucidates the previously unknown ubiquitination pathway in the development of atherosclerosis. In this pathway, under conditions that lead to atherosclerosis, the expression of MDM2 is induced, and MDM2 functions as a ubiquitin E3 ligase to target RXRβ for degradation, which contributes to mitochondrial damage and subsequently mitochondrial-related inflammation in atherosclerosis (Figure 10). With these newly acquired mechanistic insights, we propose that targeting the MDM2-RXRβ regulatory axis could represent a novel strategy for human atherosclerosis therapy.

## Data Availability

Not applicable.

## Supplementary Materials

The following supporting information can be downloaded at: https://www.mdpi.com/article/10.3390/ijms23105766/s1.
