# Supplementary material for: MDM2-Mediated Ubiquitination of RXRβ Contributes to Mitochondrial Damage and Related Inflammation in Atherosclerosis
Source: Int J Mol Sci. 2022 May 21;23(10):5766. doi: 10.3390/ijms23105766 (PMC9145909; doi:10.3390/ijms23105766)
Supplement: Supplementary file 1 [file ijms-23-05766-s001.zip › Supplemental Materials and Methods.pdf]

Supplemental Materials and Methods

2.1. Reagents.

Ox-LDL (Cat. No. YB-002) was obtained from Yiyuan Biotechnologies (Guangzhou, China). JNJ-26854165 (JNJ-165) (Cat. No. S87025) was purchased from Yuanye Bio-Tech (Shanghai, China). Cycloheximide (CHX, Cat. No. 508739), chloroquine (Cat. No. C6628), 3-methyladenine (3-MA, Cat. No. M9281) were purchased from Sigma (Sigma-Aldrich). MG132 (Cat. No. ab141003) was obtained from Abcam Inc. (Cambridge, MA, USA).

2.2. Cell culture

Human aortic endothelial cells (HAECs) were obtained from ScienCell Research Laboratories (Cat. No. #6100, Carlsbad, USA) and grown in an endothelial cell medium supplemented with endothelial cell growth supplement (Cat. No. #1001, ScienCell Technologies Inc, Carlsbad, USA) and 10% fetal bovine serum (FBS, Cat. No. SH30084.03, Hyclone, Logan, USA) according to the supplier's instructions. A total of three different batches were used. Each batch consists of HAECs from one single donor. HAECs at 3 to 8 passages were used in the study. HEK293 cells purchased from the American Type Culture Collection (Cat. No. CRL-1573, Manassas, VA, USA) were grown in DMEM supplemented with 10% FBS. The cell cultures were maintained at 37 °C under 5% CO_2 atmosphere and were routinely subcultured at 2- or 3-day intervals. When transfected with two or more plasmids, HAECs have

relatively low transfection rates. In these experiment, HEK293 cells were used for co-transfection. HEK293 cells were also used to detect the exogenous interaction between MDM2 and RXRβ.

2.3. Animal experiment

Experimental protocols were approved by the Animal Care and Use Committee of Nantong University (approval no. S20210301-012). To avoid the interference of the hormonal disturbances/changes, male mice were used in the present study. In brief, the 8-week old male $LDLr^{-/-}$ mice were randomly separated into control, high fat diet (HFD) and JNJ-165-treatment group. The control group was fed a standard chow diet for 12 weeks. In HFD group, the mice were fed a high fat diet containing 0.21 % cholesterol and 21 % fat (Cat. No. D12079B, SYSE Biotec Co., Ltd, Changzhou, China) for 12 weeks continuously. In JNJ-165-treatment group, mice were administered the same HFD for 12 weeks and dosed daily via oral gavage with 20 mg/kg/day JNJ-165 by weight for the last 4 weeks. JNJ-165 was suspended in 0.5% carboxymethyl cellulose (Cat. No. A3438, Kangruina biotechnology Co. Ltd, Beijing, China). Mice in control and HFD-only group received the same volume of 0.5% carboxymethyl cellulose vehicle gastrically. At the end of 12-week period, all mice were sacrificed under pentobarbital sodium (Cat. No. BCP07810, Biochempartner biotechnology Co. Ltd, Shanghai, China) anesthesia (50 mg/kg, i.p.). Atherosclerotic lesion severity was assessed by both en face

analysis of the aorta and by serial sections from the aortic root. Cryosections of thoracic aorta were used for immunofluorescence staining with indicated antibodies and aorta tissues were collected for further analysis such as real-time PCR and western blot. All of the methods have been reported in our previous studies [14, 21].

2.4. Quantification of atherosclerosis and aortic expressions of RXRβ

Atherosclerotic lesion severity was assessed by both en face analysis of the aorta and by serial sections from the aortic root. In brief, the entire aorta including the subclavian right and left common carotid arteries was opened longitudinally, and fixed with 4% paraformaldehyde and then stained with Oil Red O (Cat. No. O0625, Sigma, St. Louis, MO). To determine the sinus lesions in aortic root, 8 μm frozen sections of the aortic root with the presence of the aorta valve cups were prepared and stained with Oil Red O solution. The mean was calculated on 3 independent sections per mouse from 6 different mice in each group. 8 μm frozen sections of thoracic aorta were used for immunofluorescence staining with antibody against RXRβ. The mean was calculated on 3 independent sections per mouse from 6 different mice in each group.

2.5. Transfection

MDM2 adenovirus vector (Ad-MDM2) were designed and synthesized by

Hanbio Biotechnology Co., Ltd (Shanghai, China). The full-length human MDM2 sequence (GenBank number: NM_002392.5) was synthesized and cloned into the adenoviral vector pHBAd-EF1-MCS-3flag-CMV-EGFP-△loxp. Recombinant adenovirus plasmid was constructed by homologous recombination with the adenoviral backbone plasmid pAdEasy-1 in E.coli BJ5183 cells, which was linearized by Pac I and transfected into Ad293 cells for package to generate the recombinant adenovirus Ad-MDM2. MDM2 siRNA and p53 siRNA was purchased from GenePharma RNAi company (Shanghai, China). RXRβ siRNA was designed and synthesized by Hanbio Biotechnology Co., Ltd (Shanghai, China). MDM2 siRNA forward (F) primer sequence is 5'-GGCCAGUAUAUUAUGACUATT-3', and reverse (R) primer sequence is 5'-UAGUCAUAAUAUACUGGCCTT-3'. p53 siRNA forward (F) primer sequence is 5'-GUUCAAGACAGAAGGGCCUTT-3', and reverse (R) primer sequence is 5'-AGGCCCUUCUGUCUUGAACTT-3'. RXRβ siRNA forward (F) primer sequence is 5'-GCAAACGGCUAUGUGCAAUTT-3', and reverse (R) primer sequence is 5'-AUUGCACAUAGCCGUUUGCTT-3'. HA-tagged ubiquitin, Myc-tagged-RXRβ, Flag-tagged MDM2 and Flag-tagged MDM2 C464A mutant plasmids using the pCDNA3.1-CMV vector were constructed by Hanbio Biotechnology Co., Ltd (Shanghai, China). All expression plasmids were driven by the CMV promoter and encoded the corresponding human proteins. For siRNA transfection, cells were transfected with MDM2 siRNA, RXRβ siRNA or p53 siRNA for 24 h at a final concentration of 100 nM as we

described previously [14, 21]. To overexpression MDM2, cells were transduced with MDM2 adenovirus vector. MDM2 adenovirus vector diluted to MOI value of 100 were added to the culture medium for 24 h as we described previously [21]. For plasmids transfection, all plasmids were transfected with Lipofectamine 3000 (Invitrogen, San Diego, USA) for 48 h.

2.6. Analysis of inflammatory cytokines

The level of inflammatory cytokines TNF-α (Cat. No. PT518), IL-6 (Cat. No. PI330) and IL-1β (Cat. No. PI305) in cell culture supernatants were detected by ELISA kits following the manufacturer's protocols (Beyotime biotechnology Co. Ltd, Jiangsu, China).

2.7. NF-κB Transcription Factor Assay

NF-κB (p65) transcription factor DNA-binding assay was performed using a transcription factor assay kit from Cayman Chemicals (Cat.No.10007889) following the manufacturer’s protocol. Briefly, equal amounts of nuclear extracts were loaded onto coated wells provided with the kit. The primary and secondary antibodies were added and incubated for 1 h. The developing solution was added, and the antigen-antibody complex formation was recorded at absorbance 450nm.

2.8. Determination of mitochondrial parameters

Mitochondria-derived reactive oxygen species (mtROS), mitochondrial membrane potential (ΔΨm) and cytosolic mitochondrial DNA (mtDNA) were detected in HAECs in line with our previous reports [14, 21].

For measurement of mtROS, cells were seeded in 96-well plates. HAECs were transfected with RXRβ siRNA or p53 siRNA for 24 h and then treated with or without JNJ-165 (10 μM). After 1 h, the cells were stimulated with 100 μg/mL ox-LDL for additional 24 h, and then incubated with 5 μM mitoSOX Red (Cat. No.M36008, Thermo Fisher Scientific, Rockford, IL, USA). After 10 min, the cells were washed three times with warm buffer. The fluorescence intensity was measured at an emission wavelength of 510 nm and an excitation wavelength of 580 nm using a micro plate reader (synergy H1, BioTek, USA). The representative images of mtROS were visualized with a fluorescence microscope (Olympus, Tokyo, Japan).

For detection of cytosolic mtDNA, cytosolic fractions were prepared from cells with a Mitochondria Isolation Kit for Cultured Cells (Cat. No. 89874, Thermo Fisher Scientific, Rockford, IL, USA) according to the manufacturer's instructions. Briefly, cells were scraped from the culture plates and were pelleted by centrifuging the harvested cell suspensions in tubes. According to the manufacturer’s protocol, mitochondrial isolation reagents A, B, and C were added to the cell pellets and then mixed thoroughly by several inversions. The

samples were centrifuged at 700×g for 10 min at 4°C. The supernatants were then transferred to new tubes and centrifuged at 12000×g for 15 min at 4°C. The supernatants (cytosolic fractions) were collected. The cytosolic fractions were first extracted with a phenol/chloroform/isoamyl alcohol mixture (25:24:1, v/v/v) to remove protein contaminants; then, the DNA in the cytosol was precipitated with 100% ethanol. Same amount of DNA (100 ng) was applied to PCR using SYBR® Premix Ex TaqTMII (RR820A, Takara, China). MtDNA copy number was measured by quantitative PCR and normalized to nuclear DNA levels in a ratio of cytochrome C oxidase subunit III DNA over nuclear DNA (18S rRNA). The primers used were listed in supplemental Table 1.

Supplemental Table 1. Primer sequences of genes selected for analysis mtDNA

Gene		Primer sequence
cytochrome C oxidase subunit III	Forward	5′- ATGACCCACCAATCACATGC -3′
	Reverse	5′- ATCACATGGCTAGGCCGGAG -3′
18S	Forward	5′- TAGAGGGACAAGTGGCGTTC -3′
	Reverse	5′- CGCTGAGCCAGTCAGTGT-3′

For measurement of mitochondrial membrane potential (ΔΨm), The loss of ΔΨm was determined via the fluoroprobe (JC-1). Briefly, after the indicated treatments, the cultured cells were incubated with an equal amount of JC-1

(Cat. No. C2006, Beyotime Biotech, China) staining solution for 20 minutes at 37˚C. Then, the cells were placed in fresh serum-free medium, and ΔΨm was determined using a micro plate reader (synergy H1, BioTek, USA) at 490 nm excitation and 530 nm emission for green fluorescence or at 525 nm excitation and 590 nm emission for red fluorescence. The ΔΨm is the ratio of red fluorescence intensity to green fluorescence intensity, and hence, the data are expressed as a relative percent of control group. The representative images of ΔΨm were visualized with a fluorescence microscope (Olympus, Tokyo, Japan).

2.9. Quantitative real-time PCR (qRT-PCR)

qRT-PCR analysis was performed as our previous studies [14, 21]. Primers sequences used were as follows:

Supplemental Table S1. Primer sequences of genes selected for analysis mtDNA

**Gene**		**Primer sequence**
RXRβ (mouse)	Forward	5′- CTTCCCAGTCATCAGTTCTTCCA -3′
	Reverse	5′- TAAACCCCATAGTGCTTGCCTG -3′
RXRβ (human)	Forward	5′- CCTTTCCAGTCATCAGTTCTTCC -3′
	Reverse	5′- ATGTAAGGTCTTTGCGGATGGT -3′
MDM2 (human)	Forward	5′- GAATCATCGGACTCAGGTACATC -3′
	Reverse	5′- TCTGTCTCACTAATTGCTCTCCT -3′

TNF-α (human)	Forward	5′- GTCACTCATTGCTGAGCCTCT -3′
	Reverse	5′- AGCTTCTTCCCACCCACAAG -3′
IL-6 (human)	Forward	5′-GCCGCCCCACACAGACA-3′
	Reverse	5′-CCGTCGAGGATGTACCGAAT-3′
IL-1β (human)	Forward	5′-CACGATGCACCTGTACGATCA-3′
	Reverse	5′-GTTGCTCCATATCCTGTCCCT-3′
TNF-α (mouse)	Forward	5′-CGTCAGCCGATTTGCTATCT-3′
	Reverse	5′-CGGACTCCGCAAAGTCTAAG-3′
IL-6 (mouse)	Forward	5′-TGGAGTCACAGAAGGAGTGGCTAAG -3′
	Reverse	5′-TCTGACCACAGTGAGGAATGTCCAC -3′
IL-1β (mouse)	Forward	5′-ATGAGAGCATCCAGCTTCAA-3′
	Reverse	5′-TGAAGGAAAAGAAGGTGCTC-3′
β-actin (human)	Forward	5′- CATGTACGTTGCTATCCAGGC -3′
	Reverse	5′- CTCCTTAATGTCACGCACGAT -3′
β-actin (mouse)	Reverse	5′- GGCTGTATTCCCCTCCATCG -3′
	Forward	5′- CCAGTTGGTAACAATGCCATGT-3′

Experimental cycle threshold values were normalized to β-actin.

2.10. Western blot and immunoprecipitation analysis

Western blot and immunoprecipitation analysis were determined as we

described previously [14, 21]. The Whole cell extracts from tissue and cultured cells were lysed using NP-40 Lysis Buffer kit (Cat. No. P0013F, Beyotime Co, Jiangsu, China) containing 1% NP-40. Protein concentration was determined using a BCA Protein Assay Reagent (Cat. No. P0012, Beyotime Co, Jiangsu, China) according to the manufacturer's manual. For western blot, equal amounts of protein (20 μg protein of cultured cells and 30 μg protein of tissue) were separated by SDS-PAGE (6% for ABCA1, 8% for TLR9, NLRP3, PGC1α and ABCG1, 10% for MDM2, RXRβ and p53) and transferred to nitrocellulose membranes. After blocking with 5% (w/v) skimmed milk, the membrane was immunoblotted with antibody (Detailed in Supplementary Table 2) and incubated with the appropriate IRDye 680RD secondary antibodies (Cat. No.680RD, LI-COR Biosciences, Inc, Lincoln, NE, USA). The odyssey infrared imaging system (LI-COR Biosciences, Inc.) was used to detect and quantitate the blot. For immunoprecipitation, cellular extracts were incubated with the described antibodies for overnight, followed by 2-hour incubation with protein G plus-agarose (Cat. No. P2053, Beyotime biotechnology Co. Ltd, Jiangsu, China) at 4°C. Immunoprecipitated products were washed with immunoprecipitation buffer before resolved by SDS-PAGE. Proteins were detected with the indicated antibodies.

Supplemental Table S2 Antibodies for Western blotting

Antibody	Company	Country	Cat. No.
RXRβ	ABCAM	USA	ab221115

MDM2	ABCAM	USA	ab38618
TLR9	ABCAM	USA	ab37154
TLR9	ABCAM	USA	ab62577
p53	ABCAM	USA	ab26
Myc-tag	ABCAM	USA	ab9106
Flag-tag	ABCAM	USA	ab18230
NLRP3	Cell Signaling	USA	#15101
Caspase-1	ABCAM	USA	ab207802
Caspase-1	ABCAM	USA	ab1872
PGC1α	ABCAM	USA	ab54481
Ub	Cell Signaling	USA	#3936
GAPDH	ProteinTech	China	10500-1-AP

The legend for Supplemental Figure S1

Supplemental Figure S1 Effect of JNJ-165 on the protein expression of p53.

HAECs were transfected with control siRNA (con siRNA), p53 siRNA for 24 h, followed by JNJ-165 (10 μM) for additional 24 h. Protein expression of p53 was measured by western blotting. Results are presented as the mean ± SD. n=6. *$p < 0.05$ vs. control group, △$p < 0.05$.
