# Supplementary figures and images for: MDM2-Mediated Ubiquitination of RXRβ Contributes to Mitochondrial Damage and Related Inflammation in Atherosclerosis
Source: Int J Mol Sci. 2022 May 21;23(10):5766. doi: 10.3390/ijms23105766 (PMC9145909; doi:10.3390/ijms23105766)

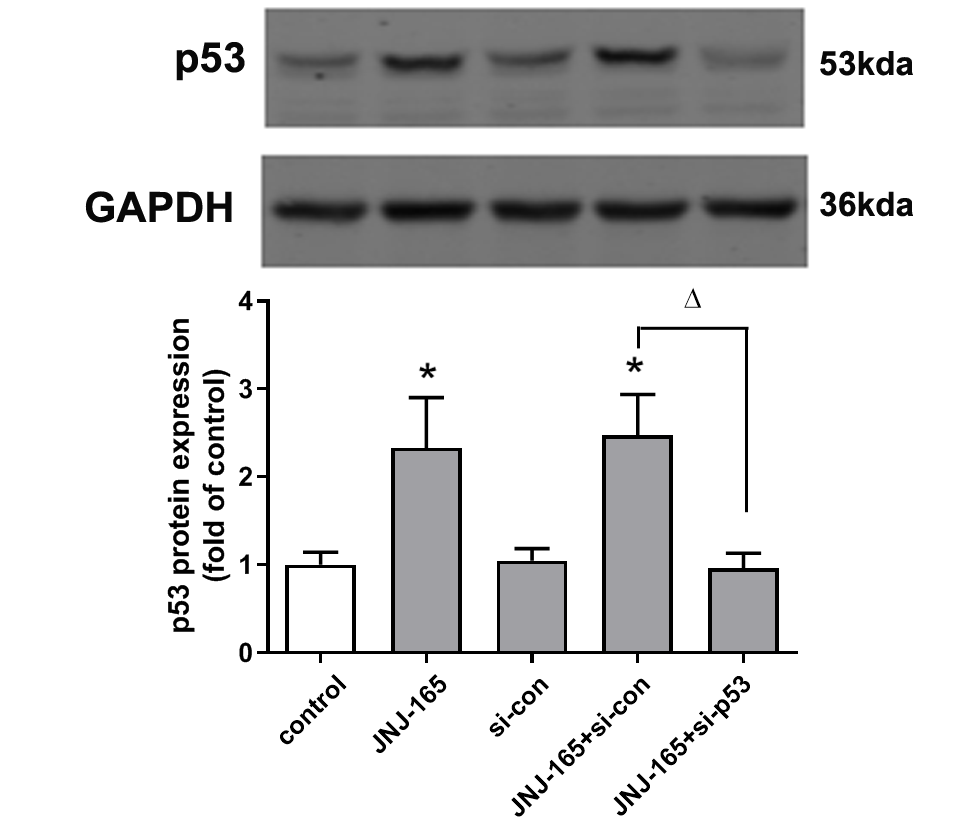

Supplement: Supplementary file 1 [file ijms-23-05766-s001.zip › Figure S1.tif]
